# Mortality Due to Cystic Fibrosis over a 36-Year Period in Spain: Time Trends and Geographic Variations

**DOI:** 10.3390/ijerph16010119

**Published:** 2019-01-04

**Authors:** Ana Villaverde-Hueso, Germán Sánchez-Díaz, Francisco J. Molina-Cabrero, Elisa Gallego, Manuel Posada de la Paz, Verónica Alonso-Ferreira

**Affiliations:** 1 Institute of Rare Diseases Research (IIER), Instituto de Salud Carlos III, 28029 Madrid, Spain; anavillaverde@isciii.es (A.V.-H.); g.sanchez@externos.isciii.es (G.S.-D.); mposada@isciii.es (M.P.d.l.P.); 2 Centre for Biomedical Network Research on Rare Diseases (CIBERER), 28029 Madrid, Spain; 3 Department of Geology, Geography and Environmental Sciences, University of Alcala, 28801 Alcalá de Henares, Spain; 4 Department of Preventive Medicine, Complejo Hospitalario Universitario de Albacete, 02006 Albacete, Spain; fmolinac@sescam.jccm.es; 5 Department of Preventive Medicine, Hospital Universitario Infanta Leonor, 28031 Madrid, Spain; elisa.gallego@salud.madrid.org

**Keywords:** cystic fibrosis, population-based mortality, mortality trends, geographic analysis

## Abstract

The aim of this study is to analyze population-based mortality attributed to cystic fibrosis (CF) over 36 years in Spain. CF deaths were obtained from the National Statistics Institute, using codes 277.0 from the International Classification of Diseases (ICD) ninth revision (ICD9-CM) and E84 from the tenth revision (ICD10) to determine the underlying cause of death. We calculated age-specific and age-adjusted mortality rates, and time trends were assessed using joinpoint regression. The geographic analysis by district was performed by standardized mortality ratios (SMRs) and smoothed-SMRs. A total of 1002 deaths due to CF were identified (50.5% women). Age-adjusted mortality rates fell by −0.95% per year between 1981 and 2016. The average age of death from CF increased due to the annual fall in the mortality of under-25s (−3.77% males, −2.37% females) and an increase in over-75s (3.49%). We identified districts with higher than expected death risks in the south (Andalusia), the Mediterranean coast (Murcia, Valencia, Catalonia), the West (Extremadura), and the Canary Islands. In conclusion, in this study we monitored the population-based mortality attributed to CF over a long period and found geographic differences in the risk of dying from this disease. These findings complement the information provided in other studies and registries and will be useful for health planning.

## 1. Introduction

Cystic fibrosis (CF) is a genetic disease with autosomal recessive inheritance that affects various organ systems [1]. Its effects on the pulmonary system are some of the most important causes of morbidity and mortality [2]. It is manifested, amongst other symptoms, by pancreatic insufficiency, chronic lung disease, infertility, and an increase in electrolyte concentration in sweat. CF is diagnosed in both sexes, although male patients are reported to have longer life expectancy [3]. It is a chronic disease affecting above all children and young adults. However, the advances that have been made in the treatment in recent decades have greatly increased life expectancy and a lot of today’s patients are adults [1,4,5].

Due to its low prevalence, CF is considered a rare disease (prevalence below 5 per 10,000 in the European Union). It occurs most frequently in Europe, North America, and Australia, and prevalence is lower in East Asia and Africa [4,6]. According to a study of CF in Europe, it has a prevalence in Spain and Italy of 0.55 per 10,000 inhabitants, while the prevalence in France and Germany is slightly higher [7]. The highest rates in Europe are in the United Kingdom and Ireland with 1.37 and 2.98 per 10,000 inhabitants, respectively, while countries in Eastern Europe, such as Latvia and Lithuania, have lower rates (1.04 and 1.30 per 10,000 inhabitants, respectively) [7].

The mortality trends have been analyzed at country and large-region level (United Kingdom, Italy, Australia, Japan, Europe, North America, and South America). In general, these studies reported a decline in mortality rates over the different periods analyzed with some variations between the different geographical areas [8,9,10,11,12,13,14]. The analysis of the studies of mortality in cohorts of patients with CF based on historical records included extensive series such as those produced by the CF Foundation in the U.S., the European CF Society, and data from official registries in other countries (Sweden, Canada, Italy, France, United Kingdom, Germany) [11,15,16,17]. In Spain, a study was conducted into mortality due to CF in children and young people under 30 years of age [18], but so far there have been no studies of spatial variability in the risk of death. Geographical analysis is particularly useful for creating hypotheses regarding exposure to environmental factors that might influence the appearance of complications in CF [19].

The methodologies applied in the available studies of mortality and survival of CF patients were based on either death certificates [8,18] or registries [16,17,20]. Studies based on patient registries, as well as indicating mortality trends, allow demographic and clinical factors to be analyzed [17,21]. However, some registries may not cover all the cases and their representativeness depends both on the number of institutions that supply them with information and the proportion of patients with CF in each institution [15]. According to the latest report on the registry of patients with CF in Spain, coverage is about 70% and there are 22 participating institutions [22]. In contrast to the studies based on patient registries, the official mortality records cover the whole population and allow us to carry out detailed analyses based on the place of residence of those who died. In this sense, the national mortality records are useful for epidemiological surveillance, as they provide data about the number of deaths directly attributed to a particular disease. They also report the total number of deaths in a region (population-based mortality) and follow a standardized methodology over time. In the specific case of rare diseases such as CF, population-based data are particularly important in order to build a series of cases that is sufficiently large as to enable a detailed temporal and geographical analysis to be performed [18,23]. In spite of the limitations of mortality studies, they continue to be a useful source of information for obtaining population-based epidemiological indicators [8,18,23].

The objective of this study is to broaden existing information about CF by means of a population-based analysis of the mortality attributed to this rare disease over a period of 36 years (1981 to 2016) and to identify the geographical variations within Spain in the risk of dying from this disease.

## 2. Materials and Methods

On the basis of the annual death records issued by the Spanish National Statistics Institute (NSI), we selected the deaths in which cystic fibrosis was cited as the underlying cause of death. We used code 277.0 from the International Classification of Diseases (ICD) ninth revision (ICD9-CM) for the period 1981–1998, and code E84 from the tenth revision (ICD10) for the period 1999–2016. For each deceased person, we considered their date of death, municipality of residence, sex, and date of birth. We also obtained the population data, broken down by sex, age, and municipality of residence.

We then calculated the specific mortality rates by age groups and sex. These were obtained for the whole study period and for each ICD period. The age-adjusted mortality rates were calculated for males, females, and both sexes. Mortality rates were expressed per 100,000 inhabitants and the European standard population was used as a reference for the adjustment. Time trends were evaluated using joinpoint regression models including two possible points of change [24]. The annual fluctuations were smoothed using the T4253H non-parametric procedure, available in SPSS 22.0 (IBM Corp, NY, USA). This procedure is based on the use of running medians to summarize overlapping segments [25].

For the geographical analysis, the municipalities were grouped into districts in order to increase the stability of the results [23]. The standardized mortality ratios (SMRs) were then calculated by district for the period 1999–2016 taking the Spanish population as a reference. On the basis of confidence intervals (CI) of 95% for each SMR, we identified the districts that showed values that were significantly higher or lower than expected compared to the total for Spain as a whole (SMR = 1.00). The SMRs were smoothed according to the model proposed by Besag, York, and Mollié, which takes into account the heterogeneity of each one [26]. For each district we also obtained the posterior probability (PP) value associated with each smoothed SMR. PP values of less than 0.20 indicate that the risk of death is lower than expected, while those greater than 0.80 indicate a higher than expected risk.

The statistical analyses were performed with Stata (StataCorp, College Station, TX, USA), Joinpoint (National Cancer Institute, Bethesda, MD, USA) and the INLA R 3.3.1 software package (Norwegian University of Science and Technology, Trondheim, Norway). ArcGIS software (Esri, Redlands, CA, USA) was used for cartographical representations.

## 3. Results

**Figure 1:**
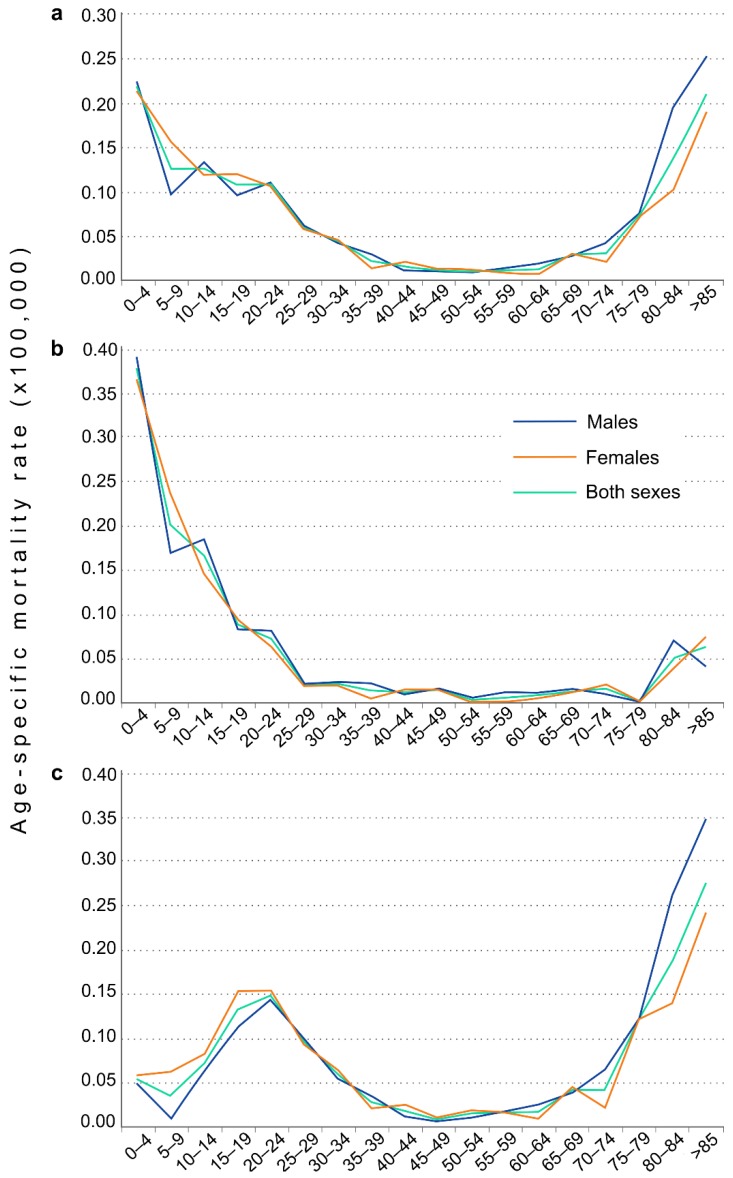
Age-specific mortality rates due to cystic fibrosis in females, males, and both sexes for 5-year age groups. (**a**) Whole period, (**b**) 1981–1998, (**c**) 1999–2016.

We identified 1002 deaths in which CF was cited as the main cause of death between 1981 and 2016 in Spain. The deaths were equally balanced between sexes in that 49.5% were males (496 of those who died) and 50.5% females (506 of those who died). The average age of death for the whole 36-year period was 27.7 years old (CI 95% 26.0–29.4), although this masks important differences between the two halves of the study period. In the first half of the study period (1981–1998) the average age of death was 13.7 years (CI 95% 12.2–15.3), while in the second half (1999–2016) it was much higher at 41.1 years (CI 95% 38.8–43.8) (*p* < 0.001). The average age of death increased significantly by 9.54% per year until 2003 (*p* < 0.001), and since then has remained unchanged. The age-specific mortality rates were highest in under-25s and over-75s, with maximums in the 0–4 years age group and the over-85s (Figure 1a). In the second half of the study period (1999–2016) there were decreases in the specific rates of child mortality and an increase in the mortality of over-75s (Figure 1b,c).

**Figure 2:**
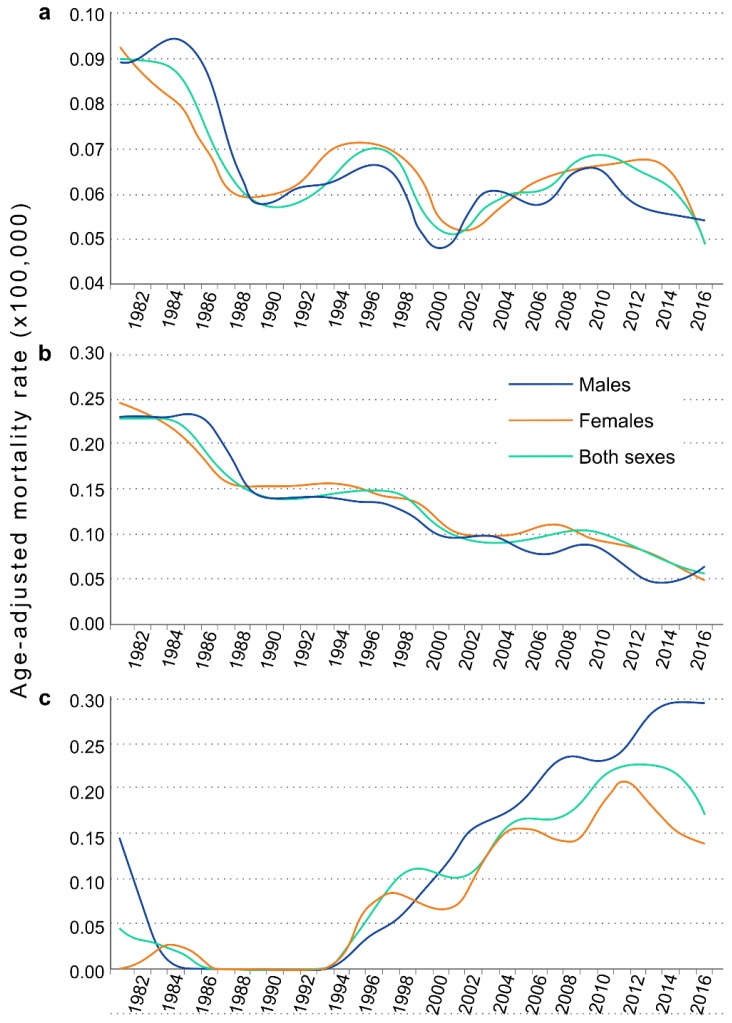
Changes in the smoothed age-adjusted mortality rates attributed to cystic fibrosis for the period 1981–2016. (**a**) All ages, (**b**) under-25s, (**c**) over-75s.

In Table 1 we present the age-adjusted mortality rates for the 36-year period. Overall, we noted a slight fall over time in the number of deaths due to CF with an annual percentage change of −0.95% (*p* < 0.001). In males, there was a fall of −1.37% (*p* < 0.01), while in women the annual change was not significant (−0.47%, *p* = 0.249) (Figure 2a). As can be seen in Figure 2b, mortality in under-25s fell by 3.77% per year between 1981 and 2016 in males (*p* < 0.01), and 2.37% in females (*p* < 0.01). By contrast, was an increase over time in mortality due to CF of 3.49% per year amongst the over-75s (*p* < 0.001). By sexes, the mortality in this age bracket increased by 2.14% in males (*p* < 0.01) and 2.55% in females (*p* < 0.01) (Figure 2c).

As regards the geographic variability, Table 2 shows the districts with SMRs that were significantly different from expected for Spain over the period 1999 to 2016 (SMR = 1.00). We identified just one district (Área Metropolitana de Madrid) with a lower mortality rate due to CF than expected and 11 districts with a higher SMR than expected, most of which were located in the south of the Iberian Peninsula. The spatial analysis shows less geographic variability when the sexes are considered separately. In males the higher than expected mortality rate was only significant in five districts, while in females it was significant in three districts.

**Figure 3:**
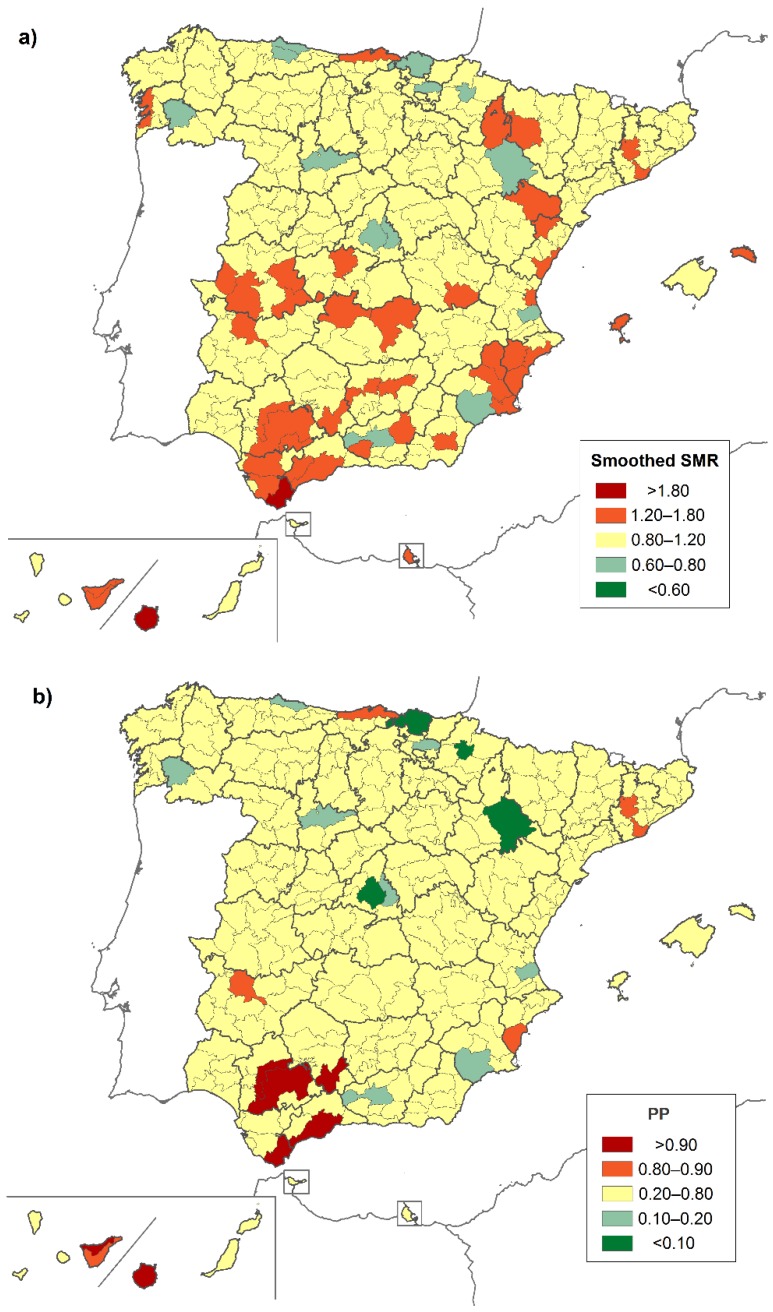
(**a**) Smoothed-standardized mortality ratios (SMRs) and (**b**) posterior probability (PP) due to cystic fibrosis by district. Both sexes, 1999–2016.

**Figure 4:**
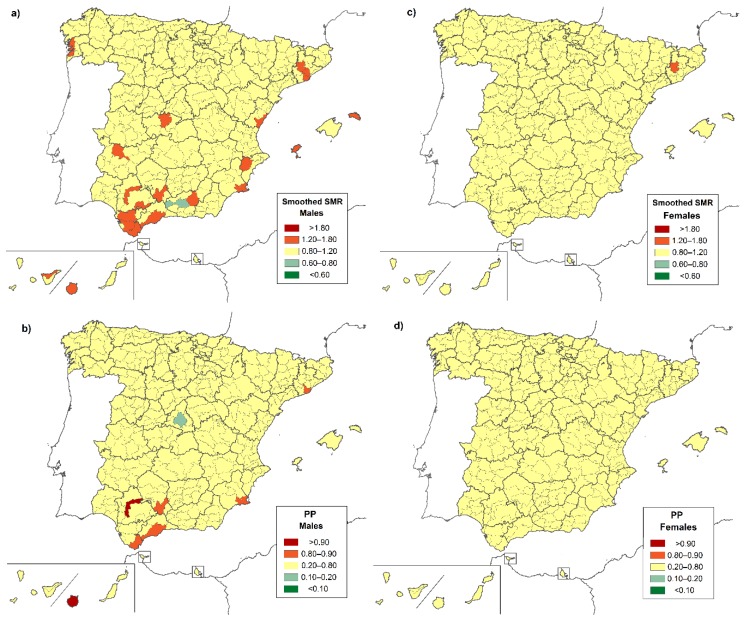
Smoothed-SMRs for cystic fibrosis by district: 1999–2016. (**a**) Smoothed-SMRs, males; (**b**) PP of smoothed-SMRs, males; (**c**) smoothed-SMRs, females; (**d**) PP of smoothed-SMRs, females.

When the information from the adjacent districts was taken into account, as reflected by the smoothed-SMRs and the PPs (Figure 3a,b), the results were consistent with a higher risk of death from CF in certain districts in Córdoba (Campiña Alta), Cádiz (Campo de Gibraltar), Sevilla (La Vega and La Campiña), Málaga (Guadalhorce), Santa Cruz de Tenerife (Norte de Tenerife), and Las Palmas (Gran Canaria). We also detected five new locations: Cantabria (Costera), Barcelona (Bages y Bajo Llobregat), Alicante (Meridional), and Badajoz (Mérida). According to Figure 4a,b, the districts with significantly higher risk for males were above all in provinces in the south, such as Córdoba (Campiña Alta), Cádiz (Campo de Gibraltar), Sevilla (La Vega), and Málaga (Centro-Sur U Guadalorce), and in certain districts in Barcelona (Bajo Llobregat) and Murcia (Campo de Cartagena). In the case of women, the geographic differences in the risk of dying were less pronounced when neighboring regions were taken into account (Figure 4c,d).

## 4. Discussion

This study monitored the mortality directly attributed to CF over more than three decades in Spain. As well as using a longer monitoring period than in previous population-based studies [18], this paper offers, for the first time, detailed evidence for the whole country regarding geographic variations in mortality.

In the European study of mortality, based on data from Eurostat for the period of 1994 to 2010, a constant reduction in mortality from CF was observed for the whole group of countries and for France and Hungary [8]. As our study covers a longer period, we were able to detect the fall in mortality due to CF over time in Spain, even though there were no significant changes in the trend over the period 1981–2016. The decline we observed in mortality in young people and the increase in over-75s confirmed the findings of other studies that produced similar results [5,17,18,27].

The average age at death also changed over the period of study, increasing significantly from 1981 to 2003 after which it remained constant. In Spain, as in other Western countries, CF is a less frequent cause of death in childhood as patients are surviving longer. As a result, the challenge is now to take care of the adults and elderly people who suffer from this disease [9,18]. The techniques used to treat the illness have improved greatly in recent decades [28]. This, together with the multidisciplinary treatment provided through specialist CF units and the improvement in the nutritional state of the population, have enabled an increase in the rates of survival and the quality of life of patients [17,29,30]. Lung transplantation is now a well-established treatment for advanced CF patients, leading to a substantial improvement in the prognosis [31,32]. Newborn screening for CF may have helped reduce mortality in many countries, although this procedure is still relatively recent in Europe and it is perhaps too early to assess its impact on CF mortality trends [8].

In this study we identified districts in which the risk of mortality from this disease is higher or lower than the expected level in Spain. Geographical variations were also identified between countries both in Europe and worldwide. In Europe, in males, higher age-standardized mortality rates were observed in Ireland and the United Kingdom, followed by Hungary and Denmark, while Sweden, Greece, and Romania had the lowest rates [8]. The situation for females was similar with higher mortality in Ireland and the United Kingdom, followed by Denmark and France, and lower in Sweden and Romania. In the United States the highest rates are in the southern regions [11], where there is a higher proportion of Hispanics. This seems to match the findings of research conducted in California that demonstrated that Hispanic patients had higher mortality rates than non-Hispanics [33]. It is interesting to note that geographic differences within the same country have also been found, for example in Canada [34]. 

As well as the variation in the distribution of mutations associated with this disease [35,36], there are environmental factors that can exacerbate CF and can perhaps explain these regional differences. It has been reported that geographical and meteorological factors can play a role in the acquisition of *Pseudomonas aeruginosa* in children with CF [19]. Malnutrition may also be related with higher levels of pulmonary compromise, a higher frequency of colonization by *Pseudomonas*, and higher mortality rates [37]. The importance of multi-resistant organisms in CF has been noted by previous researchers who reported that 86% of the representatives of the different CF units in Spain stated that multi-resistance was an urgent problem that could negatively affect the future of antimicrobial treatments and the quality of life of CF patients. Microbiological diagnosis and the detection of these pathogens in respiratory samples are essential for the correct control and monitoring of CF patients [38]. As regards the geographic distribution of alterations in the CFTR gene, the most common mutations in Spain are firstly the F508del and secondly the G542X [35,36]. The F508del mutation has been detected more frequently in northern Spain, while G542X appears more often on the Mediterranean coast and in the Canary Islands [35,36]. According to Casals et al., 70% of G542X chromosomes are from Andalusia, Murcia, Valencia, Catalonia, and the Canary Islands [35]. In this study, we detected higher mortality rates in these same regions, although they were not the only regions with high rates. Further research will be needed to assess whether our results are associated with genetic heterogeneity and/or other factors related to the seriousness of the disease.

The ability to detect the mutations associated with CF has enabled important improvements in prenatal diagnosis [39]. Newborn screening enables early intervention and, therefore, improvements in the prognosis of the disease. However, newborn screening for CF is a relatively recent innovation and it may still be too early to identify possible associated changes in mortality trends [8]. In short, the phenotype or clinical manifestations of the patients with CF are the result of the interaction of the mutations in the CFTR gene with multiple factors, some of which are environmental. This makes the disease complex or multifactorial [40]. It is also important to bear in mind that mortality does not only depend on the seriousness of the disease, but is also related to the quality of the health services and of the diagnosis. This means that further research will be necessary to discover the factors associated with the geographic variations we have identified. Future research could focus on trying to evaluate the impact of newborn screening and CFTR modifying treatments [2,41].

As regards the limitations of this study, it is possible that people who died of CF were attributed a different cause of death and that mortality was therefore underestimated [42,43]. This is why in this study we only monitored mortality attributed to CF as the underlying cause of death. The certification of death due to CF has improved over time and it is now an extremely well-characterized disease in which the diagnosis is normally well-founded and based on genetic studies. The move from ICD9 to ICD10 seems to have had little or no impact as no changes in the trends have been observed. In spite of the possible limitations, this study offers, for the first time, population-based information about the mortality directly attributed to this disease in Spain over a 36-year period. These results will help to contextualize and complement the available information in patient registries (national and international) and in population-based registries in Spain, such as the registries for rare diseases run by the Spanish regional governments and that provided by the State Registry of Rare Diseases.

## 5. Conclusions

In conclusion, mortality attributed to CF has fallen between 1981 and 2016, and the average age of death has increased considerably. Mortality in young people has fallen significantly, while it has increased in the over-75s. We have detected districts in which there is a higher- or lower-than-expected risk of mortality due to CF in Spain. However, a specific geographic pattern could not be observed and further research will be required to find out more about the causes of these geographic variations. The results of this study will increase our knowledge of this pathology, as well as provide useful information for health planning and complement the valuable information from CF registries and rare diseases registries.

## Figures and Tables

**Table 1:** Age-adjusted mortality rate (per 100,000) attributed to cystic fibrosis from 1981 to 2016.

	Both Sexes		Males		Females	
Year	Age Adjusted Mortality Rate	95% CI	Age Adjusted Mortality Rate	95% CI	Age Adjusted Mortality Rate	95% CI
1981	0.090	(0.064–0.126)	0.089	(0.054–0.154)	0.092	(0.055–0.147)
1982	0.083	(0.057–0.117)	0.076	(0.043–0.136)	0.089	(0.053–0.145)
1983	0.093	(0.066–0.130)	0.105	(0.063–0.174)	0.084	(0.049–0.138)
1984	0.086	(0.058–0.123)	0.087	(0.049–0.150)	0.085	(0.047–0.143)
1985	0.095	(0.066–0.132)	0.111	(0.069–0.177)	0.076	(0.042–0.129)
1986	0.078	(0.051–0.114)	0.098	(0.057–0.162)	0.056	(0.027–0.107)
1987	0.063	(0.039–0.096)	0.042	(0.018–0.091)	0.084	(0.047–0.143)
1988	0.056	(0.034–0.089)	0.060	(0.028–0.115)	0.052	(0.024–0.101)
1989	0.085	(0.057–0.123)	0.113	(0.069–0.178)	0.057	(0.027–0.109)
1990	0.058	(0.036–0.090)	0.037	(0.016–0.079)	0.079	(0.042–0.138)
1991	0.052	(0.030–0.085)	0.049	(0.022–0.098)	0.056	(0.025–0.111)
1992	0.062	(0.039–0.096)	0.075	(0.040–0.132)	0.049	(0.022–0.099)
1993	0.075	(0.047–0.113)	0.079	(0.041–0.139)	0.071	(0.034–0.131)
1994	0.058	(0.035–0.093)	0.040	(0.017–0.086)	0.077	(0.039–0.139)
1995	0.060	(0.038–0.094)	0.048	(0.021–0.098)	0.075	(0.040–0.132)
1996	0.089	(0.061–0.127)	0.108	(0.066–0.171)	0.067	(0.035–0.123)
1997	0.076	(0.050–0.114)	0.083	(0.045–0.144)	0.069	(0.036–0.126)
1998	0.057	(0.034–0.091)	0.044	(0.018–0.096)	0.069	(0.035–0.128)
1999	0.065	(0.042–0.099)	0.056	(0.028–0.107)	0.074	(0.040–0.133)
2000	0.042	(0.025–0.070)	0.045	(0.022–0.091)	0.040	(0.018–0.088)
2001	0.050	(0.030–0.082)	0.049	(0.022–0.102)	0.053	(0.026–0.105)
2002	0.038	(0.022–0.065)	0.033	(0.013–0.077)	0.041	(0.019–0.088)
2003	0.070	(0.046–0.105)	0.079	(0.043–0.137)	0.063	(0.033–0.115)
2004	0.090	(0.062–0.128)	0.077	(0.043–0.132)	0.104	(0.062–0.168)
2005	0.053	(0.033–0.082)	0.054	(0.027–0.100)	0.052	(0.026–0.098)
2006	0.030	(0.016–0.052)	0.029	(0.011–0.067)	0.029	(0.012–0.067)
2007	0.064	(0.041–0.096)	0.047	(0.023–0.090)	0.087	(0.049–0.146)
2008	0.065	(0.043–0.096)	0.074	(0.043–0.123)	0.058	(0.029–0.107)
2009	0.076	(0.052–0.109)	0.068	(0.037–0.117)	0.084	(0.049–0.138)
2010	0.072	(0.048–0.104)	0.088	(0.052–0.142)	0.053	(0.027–0.098)
2011	0.055	(0.035–0.082)	0.044	(0.022–0.082)	0.067	(0.037–0.115)
2012	0.061	(0.041–0.090)	0.056	(0.030–0.099)	0.066	(0.037–0.113)
2013	0.079	(0.054–0.113)	0.057	(0.031–0.101)	0.106	(0.064–0.167)
2014	0.063	(0.040–0.094)	0.072	(0.040–0.124)	0.053	(0.026–0.100)
2015	0.057	(0.038–0.085)	0.050	(0.027–0.090)	0.066	(0.037–0.114)
2016	0.049	(0.030–0.078)	0.054	(0.027–0.100)	0.044	(0.020–0.090)

**Table 2:** Comparison of SMR values (95% CI) among districts with significant results for the period 1999–2016, for both sexes, and for males and females separately.

District	Province	Location	Both Sexes	Males	Females
Very low risk					
Área Metropolitana de Madrid	Madrid	C	0.658 (0.453–0.924)	-	0.598 (0.341–0.970)
High risk					
Brozas	Cáceres	W	15.462 (1.736–55.825)	-	30.304 (3.403–109.413)
Alto Maestrazgo	Castellón	E	12.091 (1.358–43.654)	-	-
Logrosán	Cáceres	W	10.474 (1.176–37.817)	-	-
Guadix	Granada	S	5.468 (1.099–15.978)	-	-
Campiña Alta	Córdoba	S	3.168 (1.269–6.527)	4.652 (1.499–10.856)	-
Campo de Gibraltar	Cádiz	S	2.892 (1.245–5.698)	3.783 (1.219–8.829)	-
Norte de Tenerife	SC de Tenerife	SW *	2.473 (1.184–4.549)	-	2.849 (1.040–6.200)
Gran Canaria	Las Palmas	SW *	2.331 (1.423–3.600)	2.664 (1.328–4.767)	-
La Vega	Sevilla	SW	2.201 (1.004–4.178)	2.169 (1.119–3.788)	-
La Campiña	Sevilla	SW	1.934 (1.225–2.902)	-	2.816 (1.028–6.129)
Guadalhorce	Málaga	S	1.798 (1.126–2.722)	-	-
Campo de Cartagena	Murcia	SE	-	3.395 (1.240–7.390)	-

* Island territories (Canary and Balearic Islands); CI: confidence interval; C: Centre; E: East; N: North; NE: Northeast; NW: Northwest; S: South; SE: Southeast; SW: Southwest; W: West.
